# Numerical Simulation and Experimental Study of the Drop Impact for a Multiphase System Formed by Two Immiscible Fluids

**DOI:** 10.3390/s22093126

**Published:** 2022-04-19

**Authors:** Agata Sochan, Krzysztof Lamorski, Andrzej Bieganowski

**Affiliations:** Institute of Agrophysics, Polish Academy of Sciences, Doświadczalna 4, 20-290 Lublin, Poland; k.lamorski@ipan.lublin.pl (K.L.); a.bieganowski@ipan.lublin.pl (A.B.)

**Keywords:** splash phenomenon, petroleum contamination, multiphaseInterFoam solver

## Abstract

The multiphase splash phenomenon is especially interesting in the context of environmental protection, as it could be a mechanism for transporting various types of pollution. A numerical 3D multiphase transport model was applied to a splash that occurred under the impact of a petrol drop on the water surface. The splash phenomenon in immiscible liquids was simulated using the multiphaseInterFoam solver, i.e., a part of the OpenFOAM computational fluid dynamics software implementing the finite volume method (FVM) for space discretization. Thirteen variants with a variable drop size (3.00–3.60 mm) or drop velocity (3.29–3.44 m/s) were conducted and validated experimentally based on splash images taken by a high-speed camera (2800 fps). Based on the numerical simulation, it was possible to analyse aspects that were difficult or impossible to achieve experimentally due to the limitations of the image analysis method. The aspects included the cavity spread, the jet forming moment, and, notably, the scale of the petroleum contamination spread in the splash effect. The simulations showed that droplets detaching from the crown did not consist of pure water but were mostly a “mixture” of water and petrol or petrol alone. The applied modelling workflow is an efficient way to simulate three-phase splash phenomena.

## 1. Introduction

The behaviours of the impact of drops on the free surface of a deep liquid layer can be categorised into the three main phenomena: drop rebounding [1,2,3], coalescence [4,5,6], and splashing [7,8,9]. The splashing phenomenon is frequently associated with the simultaneous formation of a crown and a cavity, whereby the formation and development of the crown may subsequently cause drop fragmentation [10], the detachment of satellite droplets [3,11], or closure of a dome [12,13], while crater formation may cause bubble entrapments [14,15], ejecting jets [16,17], and the generation of vortex rings [18,19].

Depending on the analysed systems (water, oil derivatives, single-phase, multiphase, miscible or immiscible liquids, low or high velocity drop impacts), the results obtained serve for the development of the petroleum and chemical industries, e.g., internal combustion engines, oil recovery, firefighting systems, inkjet printing, and spray cooling and painting [20,21,22], and can be used in various fields of science, from environmental protection in the case of, for example, fuel spills, pollution, and microbial transport in air and water [11,20,23], and splash erosion [24,25,26], to converting the energy of raindrops into electrical energy through proper transducers [27,28,29]. Recently, the role of these systems in the spread of COVID-19 has been investigated [30,31,32].

The multiphase splash phenomenon is especially interesting in the context of the possible implications for a diverse set of important processes. In the context of the interactions of systems of surface water resources with the atmosphere and biosphere, splash is considered by some researchers to be an unfavourable phenomenon, as it is a mechanism for transporting various types of pollution, e.g., petroleum substances [11] and biological contaminants [33,34]. These processes, especially fluid fragmentation, are also important for agriculture and food safety, where they are considered to be a spread mechanism in foliar disease transmission [31]. Therefore, it is advisable to further analyse the phenomenon, especially using numerical modelling which will make it possible to identify and describe aspects of the phenomenon that cannot be observed through experimentation; in other words, to complete the information that can be measured in the experiment.

One possible method for using different fluids in simulations is the VOF method. A literature search shows that the modelling of two-phase systems is very popular, and often open-source computational fluid dynamics (CFD) software—OpenFOAM (OF) implementing the VOF method—is used for this purpose. As a result, the OF two-phase solver (called InterFoam) has been thoroughly validated, as shown in numerous published studies [20,35,36,37,38]. On the other hand, multiphase modelling within the OF framework is performed using a different solver, i.e., multiphaseInterFoam, which is based on similar concepts and shares only a part of the code with its two-phase counterpart. There are very few studies dealing with the validation of the multiphase OF modelling solver; only modelling of the lancing of furnace tap-holes has been presented [39], and studies on stratified sloshing in a tank [40] have been published. No OF multiphase studies were related to the phenomenon of the drop impact.

Therefore, the main aim of our work was to develop and validate a numerical 3D multiphase transport model, and thus provide information on the spread of petroleum substances during the splash (this cannot be observed experimentally and the information can only be obtained from the model). The model was based on the OF CFD suite—multiphaseInterFoam was the validated solver. Since the validation of the numerical model was based on the experimental results and these results can be biased by errors which are difficult to estimate, the additional aim of this work was to assess to what extent the error in the reading of drop velocity and size may influence the results of the simulation.

## 2. Materials and Methods

### 2.1. Experiments

The systems of immiscible liquids in which the splash occurred under the impact of a single drop of petrol on the water surface were tested. This step was based on previously published results for single-phase systems [13]. The basic properties of the liquids used are shown in Table 1. Density was determined with the U-tube oscillation method according to EN ISO 12185:1996 [41], while kinematic viscosity was determined with the capillary method according to EN ISO 3104:1996 [42] in an accredited laboratory of Orlen Polska (Poland). Surface tension analysis was performed using the pendant drop method with a Drop Shape Analyzer—DSA100 (KRÜSS GmbH, Hamburg, Germany).

Measurements of the drop properties (size and velocity) and the course of the splash phenomenon were recorded using a high-speed camera Phantom MIRO M310 (Vision Research, Wayne, NJ, USA) with a speed of 2800 frames per second at the highest available resolution (1280 × 800 pixels) and Phantom Camera Control software [43]. The optical axis of the camera was at the same level as the water surface. For proper lighting of the samples, three LED panels were used (each back-lighting panel had dimensions of 0.6 × 0.6 m and a guaranteed luminous flux of 3500 lumens) as described in [44].

The drops of petrol were created using a peristaltic pump as presented in [45]. A single drop fell into the water from 0.7 m (a height corresponding to the location of the fuel tank in agricultural machines). The radius and velocity of each drop were 1.65 mm and 3.37 m∙s^−1^, respectively. Water was placed in a 195 × 195 × 100 mm glass vessel. The height of the liquid column was many times larger than the diameter of the falling drop.

The liquid system was also characterised by determining the Weber number (We)—the ratio of inertial to surface tension forces—and the Froude number (Fr)—the ratio of inertial to gravitational forces:

(1) $$We=\rho v^{2}d\sigma^{-1}$$

(2) $$Fr=v^{2}g^{-1}d^{-1}$$

where:

*ρ*—liquid density (kg∙m^−3^),
*σ*—surface tension for a fluid–air interface (N∙m^−1^),
*d*—diameter of the drop (m),
*v*—velocity of the drop (m∙s^−1^),
*g*—acceleration of gravity (m∙s^−2^).

For the above-mentioned drop values, the value of the Weber number was 986, and the Froude number was 351.

All experiments were repeated three times in controlled constant conditions: temperature 20 ± 1 °C, pressure (1000 ± 2 hPa), and humidity (50 ± 2%).

### 2.2. Image Analysis—Real (Experimental) Splash Phenomenon

**Figure 1:**
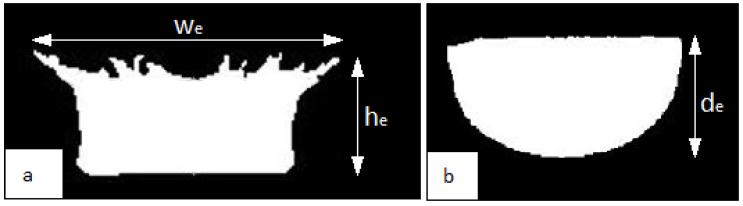
Size parameters of the experimental crown (**a**): h_e_—crown height, w_e_—external width of the crown. Size parameter of the experimental cavity (**b**): d_e_—depth of the cavity. Index “e” specifies the results from the experiment.

The images obtained with the high-speed camera were analysed in the GNU Image Manipulation Program (GIMP) 2.0 [46]. First, they were converted to black and white images. Then, points were applied indicating, among others, the highest point of the crown or the lowest point of the cavity and the level of liquid in the vessel. Next, on the basis of the coordinates of the points, the distances between particular points were determined. This analysis enabled the determination of three parameters of the primary splash forms (Figure 1), which formed the basis of comparisons with the numerical simulation:

- the experimental crown height (h_e_)—measured from the liquid surface to the highest of the crown spikes;
- the experimental external width of the crown (w_e_)—measured at the height of the crown spikes at their maximum spread;
- the experimental depth of the cavity below the water surface (d_e_)—measured from the liquid surface to the deepest point below the surface.

### 2.3. Numerical Simulations

The phenomenon of the petrol drop falling into the water pool was simulated using OpenFOAM [47] computational fluid dynamics software implementing the finite volume method (FVM) for space discretization [48]. The OF set of software tools was used for pre-processing (including mesh generation and mesh partitioning), calculations, and post-processing of the results. Simulations were conducted using the multiphaseInterFoam solver, implementing the incompressible multiphase flow of immiscible phases. The VOF method was used for the description of distinct phases. Surface tension at the interface was implemented using a continuum surface force (CSF) model together with MULES interface compression to ensure that the phase interface was as sharp as possible [49]. Five independent variables were taken into consideration: velocity—*u*, pressure—*p*, and three scalar fields indicating the presence (1) or absence (0) of: air—α₀, water—α₁, and oil—α₂ at that point of the space.

Assuming the sharp interface between the non-mixing phases, the mass and momentum conservation equations were applied to each of the *k* phases:

(3) $$\frac{\partial \alpha_{k}}{\partial t}+{\vec{u}}_{k}\cdot \nabla \alpha_{k}+\nabla \cdot \left({\vec{u}}_{c}\alpha_{k}\left(1-\alpha_{k}\right)\right)=0$$

(4) $$\frac{\partial \left(\rho_{k}\alpha_{k}{\vec{u}}_{k}\right)}{\partial t}+\left(\rho_{k}\alpha_{k}{\vec{u}}_{k}\cdot \nabla \right){\vec{u}}_{k}=-\alpha_{k}\nabla p+\nabla \cdot \left(\mu_{k}\alpha_{k}\nabla {\vec{u}}_{k}\right)+\rho_{k}\alpha_{k}\vec{g}+{\vec{F}}_{k}$$

where: *ρ_k_* is the density and *μ_k_* is the viscosity of the phase *k,* respectively. The last term of the mass conservation equation, Equation (3), is responsible for the artificial interface compression. It ensures that the interface does not smear during the interface movement. The interface compression velocity *u_c_*, i.e., the velocity normal to the interphase, is described by the following equation, where the interface compression coefficient 0 ≤ *C_α_* ≤ 1 is a scalar constant determining the strength of the interface compression:

(5) $${\vec{u}}_{c}=C_{\alpha }\left|\vec{u}\right|\frac{\nabla \alpha }{\left|\nabla \alpha \right|}$$

The momentum equation, Equation (4), involves the *F_k_* term responsible for the capillary forces:

(6) $${\vec{F}}_{k}=\sigma \kappa \nabla \alpha_{k}$$

where *σ*—the surface tension coefficient, and *κ*—the local surface curvature:

(7) $$\kappa =-\nabla \cdot \left(\frac{\nabla \alpha }{\left|\nabla \alpha \right|}\right)$$

Details of VOF implementation for multiphase modelling can be found in [49].

**Figure 2:**
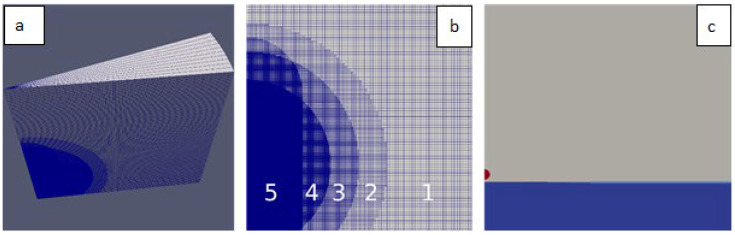
Numerical mesh used in the study: (**a**) overview of the whole wedge mesh; (**b**) cross-section through the central part (40 × 40 mm)—fragment showing refinement areas (numbered: 1, 2, 3, 4, and 5); (**c**) configuration of simulated fluid at the initial time (fluids: air—grey, water—blue, petrol—red).

In the simulations used for experimental comparisons, axial symmetry of the phenomenon was assumed. This assumption was valid if we focused on all the observed phenomenon elements (i.e., wave, crown, cavity, and jet formation) other than the formation of crown spikes and detaching satellite droplets. The experimental validation confirmed the validity of this approach. The simulated area had a wedge-like shape of 60 mm in height and 60 mm in radius (Figure 2a). The mesh was generated by rotationally extruding a 2D initial mesh. Cell refinement was applied in the mesh generation in the drop impact area (Figure 2b). The initial size of the cells was 0.6 mm, and it was twice smaller in each subsequent refinement region, reaching a final size of 0.0375 mm. The total number of cells in the mesh was 3.3 × 10^9^.

The proper mesh cell size used for mesh generation was chosen based on simulations made in advance for three different refined meshes with initial 0.8, 0.4, and 0.2 mm and minimal 0.2, 0.1, and 0.05 mm cell sizes.

Simulations are defined by initial and boundary conditions. Boundary conditions have to be defined for the top, bottom, and external parts of the mesh. For all of these regions, Neuman-type zero gradient boundary conditions were used for all the variables:

(8) $$\frac{\partial \alpha_{k}}{\partial \vec{n}}=0,\;\frac{\partial p}{\partial \vec{n}}=0,\;\frac{\partial {\vec{u}}_{k}}{\partial \vec{n}}=0$$

where $\vec{n}$ denotes the normal to the boundary.

The initial conditions defined: the geometry of the fluids (air—α₀, water—α₁, and oil—α₂) at the initial time (Figure 2c), assigning a value of one to the fluid fraction α_k_ in cells holding fluid *k* and zero otherwise; pressure field; and the initial velocity, taking into account the non-zero initial velocity of the drop *v*:

(9) $$p=0,\;{\vec{u}}_{0}=0,\;{\vec{u}}_{1}=0,\;{\vec{u}}_{2}=-v\hat{z}$$

where $\hat{z}$—a unit *z*-axis versor.

The simulations were carried out using adaptive time control where the criterion for eventual timestep reduction was the maximum acceptable value of the Courant number set to 0.45. The first 34 ms of the drop falling phenomenon were simulated according to the experimental analysis. The results were saved with a timestep of 0.357 ms, appropriate for comparisons with the high-speed camera videos.

The hardware used for numerical simulations was the seven-node cluster of two eight-core CPU, 512 GB RAM PCs connected by InfiniBand EDR infrastructure. To effectively utilise the available hardware resources, the simulations were performed in parallel using 14 mesh subdomains (two per node cluster). Scotch mesh partitioning was used for mesh domain decomposition.

The input data used to model the splash phenomenon in this work can be divided into two groups:

- liquid properties (Table 1);
- properties of the drop, i.e., its size and impact velocity.

For all the above physical quantities, preliminary tests were carried out to see the degree to which a small change in the value of each quantity induces changes in modelling results. It was found that a small overestimation/underestimation of the values of liquid properties did not result in a significant differentiation of the created models; however, in the case of the drop properties, the sensitivity of the model was greater. Therefore, this fact was taken into account in further steps by conducting a series of simulations for overestimated and similarly underestimated quantities in relation to the drop values measured from the recorded images. Such an approach is also justified by the fact that the unambiguous determination of the drop’s boundary in the image (constituting one frame from the recorded film) was subject to error because this border was usually slightly blurred.

The smallest unit in the interpretation of the individual frames of the recorded film, from which the drop’s diameter and velocity were taken, was one pixel (100 × 100 µm). Therefore, additional variants were used to the value read in the simulations, increasing the readings by one, two, and three pixels and decreasing them by the same amount. Hence, for both the drop’s properties and the measured values, six variants of each were adopted for the simulation (Table 2).

Therefore, the drop’s size in the subsequent variants of the simulation varied from 3.00 to 3.60 mm (in steps of 0.10 mm), and these variants were marked with symbols d(−3), d(−2), d(−1), d,v(0), d(+1), d(+2), and d(+3). Similarly, the drop’s impact velocity range was 3.29 to 3.44 m·s^−1^ (in steps of 0.025 m·s^−1^), and the variants of the simulation were marked with symbols v(−3), v(−2), v(−1), d,v(0), v(+1), v(+2), and v(+3).

### 2.4. Image Analysis—Simulation of the Splash Phenomenon

**Figure 3:**
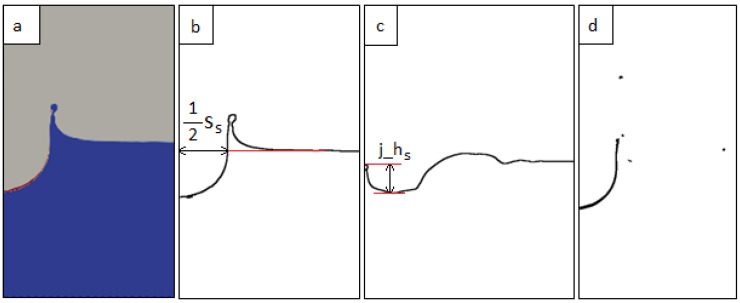
Example of a splash simulation scan from ParaView (**a**): blue—water, red—petrol, grey—air; (**b**) simulated primary splash forms: crown and cavity; (**c**) simulated secondary form: jet; (**d**) simulated distribution and dispersal of petroleum substance.

The 2D cross-sections of the simulated fluid configuration for subsequent time steps were extracted using ParaView software [50] (Figure 3a) and were analysed in GIMP. First of all, the cross-sections were scaled to match the size of the images of the real phenomenon, i.e., the size of the images from the camera. Next, the images were converted to the indexed mode with a one-bit colour palette. The next step was to return to the RGB mode, in which a given colour (e.g., background) was converted to transparency. As a result, only the line on the phase boundary remained on the images (liquid–air: Figure 3b,c, or water–petrol: Figure 3d). Then, the same procedure as that described for images of the real splash phenomenon was followed, i.e., specific points were applied to the images and the distances between them were automatically determined, obtaining the following parameters of different splash forms:

- the simulated crown height (h_s_);
- the simulated external width of the crown (w_s_);
- the simulated depth of the cavity below the water surface (d_s_);
- the simulated spread of the cavity (s_s_), measured on the liquid level (Figure 3b);
- the simulated jet height (j_h_s_), measured from the bottom of the cavity (Figure 3c).

In order to validate the developed models, the recorded films of the splash phenomenon were used, where the same moments of the duration of the phenomenon were compared. The comparison of the shape of the experimental splash forms with their simulations was presented on the basis of three parameters:

- the difference between the height of the experimental crown and its simulation (∆h);
- the difference between the external width of the experimental crown and its simulation (∆w);
- the difference between the depth of the experimental cavity and its simulation (∆d).

## 3. Results

### 3.1. Results of the Experiment

At the recording speed of 2800 frames per second, the time interval between the frames was 0.357 ms. Frame 0 (the initial time) corresponded to the condition when the falling drop touched the water surface. Since the film analysis indicated that the first pronounced changes in surface geometry were observed in Frame 5 (1.79 ms after a drop hit) and primary splash forms were no longer visible in Frame 95 (33.93 ms), this time interval was analysed by dividing it into 19 subintervals of five frames each. In general, the primary splash forms are understood as the crown and cavity formed and developed from the moment the drop hits until the first equalisation of the liquid level. The primary forms collapse, but if the drop impact is energetic enough, secondary splash forms, e.g., the central jet, secondary drop, column drops, and repeated cavity, can be created [3,5,7]. Secondary splash forms were not studied and simulated in this work. However, before the final disappearance of the crown and cavity, the jet was already beginning to form, so the results show its height up to the point when the primary forms disappeared.

**Figure 4:**
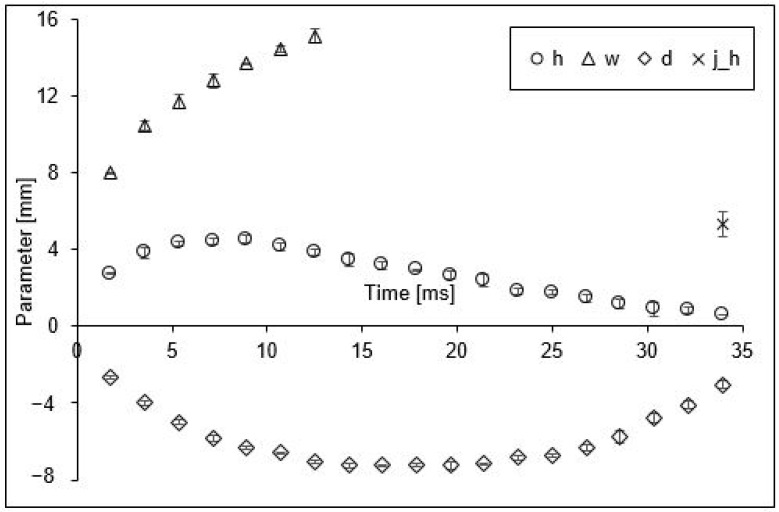
Size of the splash expressed through the parameters of the generated forms: crown height (h), external width of the crown (w), depth of the cavity below the water surface (d), and jet height (j_h), presented in subsequent moments of the splash phenomenon after the impact of a petrol drop falling from 0.7 m. The negative values of the cavity parameters are the consequence of the assumption that the water level was the reference.

The effect of the petrol drop’s impact on the water surface is presented in Figure 4. The level of the water was treated as the reference. Since the crowns were created above the surface, the parameters of their values are shown in the first quadrant of the coordinate system, i.e., they have positive values. Consequently, the values of the cavity parameter are shown in the fourth quadrant of the coordinate system, i.e., they have negative values.

**Figure 5:**
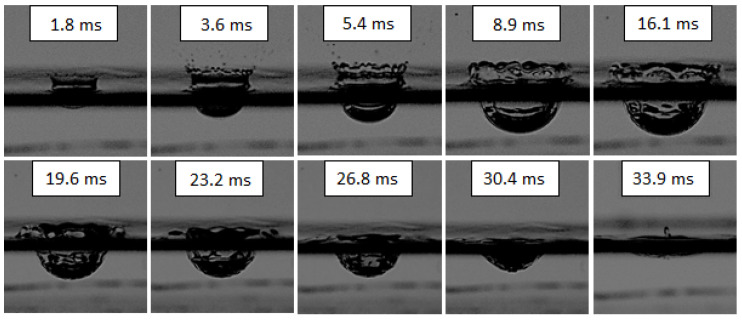
Changes in the shape of the primary forms in the course of their development and disappearance. The values in ms in the pictures refer to the time interval from the moment the drop hit the liquid surface. The dark band visible across the images was caused by the occurrence of a meniscus at the wall of the vessel.

In order to more easily understand the changes of particular quantities presented in Figure 4, it is worth analysing the shapes of the forms at the important moments of their development (Figure 5). The analysis of these changes was slightly hindered by the dark band running horizontally in each picture. This band, with an average height of about 2.5 mm, was caused by the presence of a meniscus at the walls of the glass vessel. Because the meniscus was convex, the upper limit of the belt determined the water level in the vessel, and the meniscus made it impossible to measure the crown base and the cavity width.

When the drop hit the surface of the liquid, two primary forms of splash: the crown and the cavity began to develop at almost the same time. However, both the picture (Figure 5) and the graph (Figure 4) show that the cavity initiation time was longer than that of the crown. While the crown started to fall and dissipate quickly after 8.9 ms, the greatest depth of the cavity was recorded at 17.9 ms. Differences were also observed in the way both of the forms dissipated. The crown lost its height and at the same time spread outwards until reaching the level of the liquid in the vessel. The depth of the cavity also decreased, reaching the lowest measurable value after 34 ms. Although this depth was trending towards zero, at the next time interval (36 ms), the cavity was shallower than the meniscus covering it; hence, its depth was impossible to measure.

It should be mentioned that the surface alignment was a momentary state and the liquid still had the energy to form a central jet. This secondary form is visible/measurable in the last picture in Figure 5. The jet height was measured from the bottom of the cavity, giving the value of 5.3 mm shown in Figure 4.

### 3.2. Results of Simulations

#### 3.2.1. Effect of Drop Size

**Figure 6:**
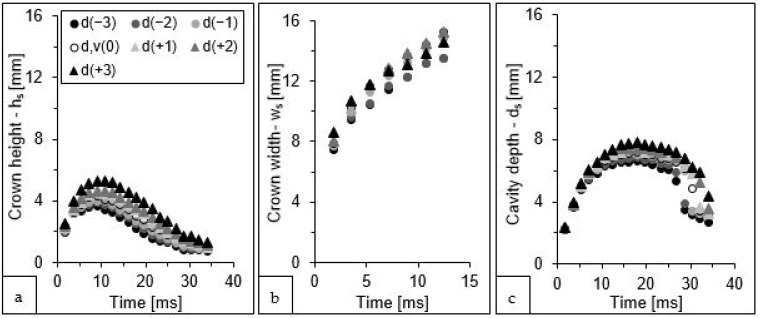
Change in the three splash parameters values: (**a**) height of the crown—h_s_; (**b**) external width of the crown—w_s_; (**c**) depth of the cavity—d_s_, presented in subsequent moments of the splash phenomenon for seven simulations differentiated by the diameter of the falling drop.

As mentioned previously, it was very difficult to measure accurately the drop size. Therefore, besides the measured value, the additional drop sizes were used for the simulations, i.e., one, two, and three pixels larger and one, two, and three pixels smaller. The differences in the crown and cavity sizes obtained from these seven simulations are shown in Figure 6.

The change in the drop size had a significant influence on the parameters of the subsequent model variants. As could be expected, the larger the drop, the larger the sizes of the crown and cavity. However, this influence changed with time. For instance, any error in the measurement of the drop size had the biggest potential to influence the crown height in the period of 7 to 23 ms (Figure 6a), where the difference between the largest and smallest height of the crown was approximately from 1.6 to 2.0 mm. The greatest diversity of subsequent model variants was observed for the crown height at 21 ms—approximately 2 mm, which accounted for as much as 57% of the maximum value. Before and after this time interval, the influence was also visible but smaller (0.3 to 1.3 mm difference between the extreme values).

Another relationship was observed for the external width of the crown (Figure 6b), for which the differences between the values generated by the successive simulation variants oscillated around 1.4 mm in the analysed time interval, which amounted to only about 11% of the maximum crown width.

**Figure 7:**
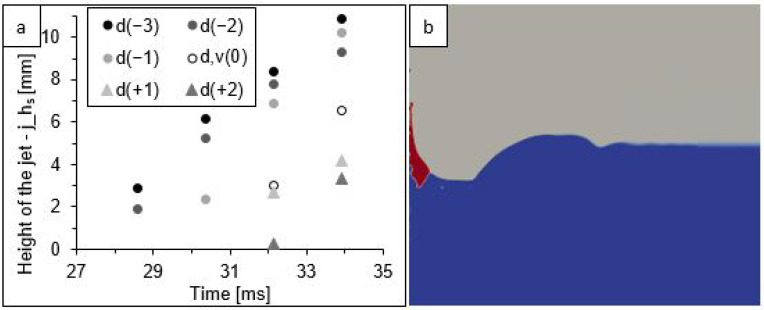
A jet: (**a**) changes in the jet height in a function of time for six models of size variants; (**b**) an example of jet simulation at 34 ms for model d,v(0)—the colours signify: water (blue), petrol (red), and air (grey).

For the depth of the cavity (Figure 6c), steadily increasing changes were observed from 0.1 up to about 3 mm, which was from approximately 5 to 50% of the maximum depth value at a given time. In addition, another interesting relationship was observed, where the values at 28 ms for the two least energetic variants (models d(−3) and d(−2)) were significantly lower than for the other simulations. At the next analysed time (30 ms), a similar effect was observed for the variants with the three smallest diameter drops, at 32 ms for five variants, and at 34 ms for six variants. This observation is undoubtedly related to the fact that, at these four specific times and for the above-mentioned simulation variants, the appearance of a jet (Figure 7), formed from the collected liquid, was recorded. This secondary form “disturbed” the course of the free dissipation of the cavity below the surface of the liquid.

#### 3.2.2. Effect of Drop Velocity

**Figure 8:**
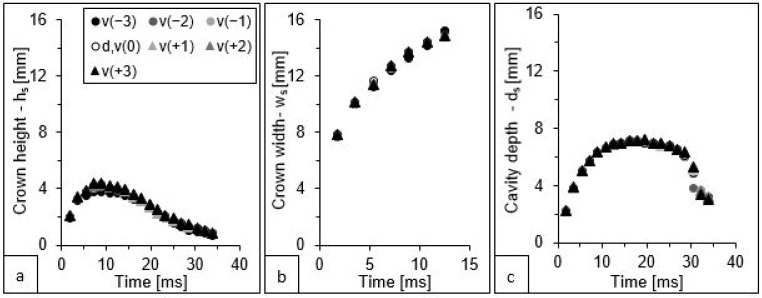
Change in the three splash parameter values: (**a**) height of the crown—h_s_, (**b**) external width of the crown arms—w_s_, and (**c**) depth of the cavity below the water surface—d_s_, presented in the subsequent moments of the splash phenomenon for seven simulations differentiated by the velocity of the falling drop.

The values of the three parameters characterising the splash generated for seven simulation variants including the variable velocity of the falling drop from model v(−3) (3.29 m·s^−1^), model d,v(0) (3.37 m·s^−1^), and model v(+3) (3.44 m·s^−1^) are presented in Figure 8.

The change in the impact velocity affected the differentiation of the analysed splash parameters to a much lesser extent than the change in the drop diameter. The graphs presented in Figure 8c show that the values of the cavity depth differed between the extreme variants—v(−3) and v(+3)—by a maximum of 8%. A slightly greater differentiation between the extreme variants was observed for the height of the crown (Figure 8a). For this parameter, the maximum difference was 28%, but it occurred only at certain moments in time and not throughout the duration of the simulation. In the case of the external width of the crown (Figure 8b), the differences between the largest and smallest values oscillated around 0.3 mm, which accounted for only about 2% of the maximum crown width at a given time.

Similarly, as in the case of the variants taking into account the change in the drop diameter, here the crown height parameter reached its maximum at 8.9 ms (Figure 8a), while the cavity depth parameter reached its maximum at 17.9 ms (Figure 8c). The parameter determining the width of the crown was characterised by a constant increase in value (Figure 8b).

**Figure 9:**
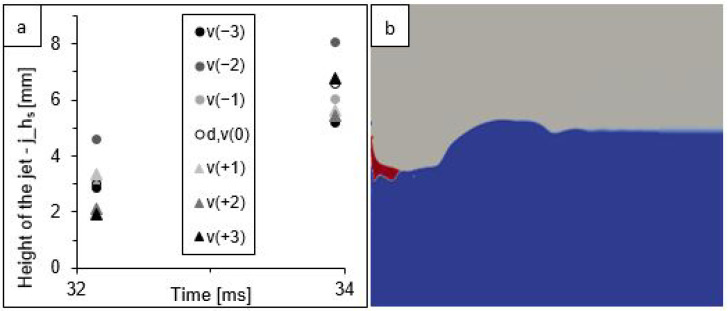
A jet: (**a**) changes in the jet height in a function of time for seven models of velocity variants; (**b**) an example of jet simulation at 32 ms for model d,v(0)—the colours signify: water (blue), petrol (red), and air (grey).

Again, for the last two analysed moments in time (32 and 34 ms), the appearance of secondary forms of splash was observed in all variants of the simulation. Figure 9 shows their height and an example of an image from the simulation variant d,v(0).

### 3.3. Model Validation

**Figure 10:**
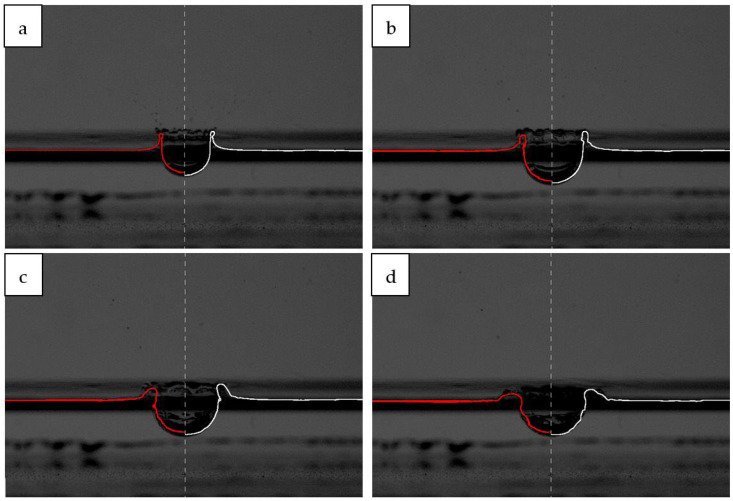
Pictures of the real splash and simulated interfacial profile from variant d,v(0)—white line and the variant d(−3)—red line, at different time intervals: (**a**) 5.4 ms; (**b**) 10.7 ms; (**c**) 16.1 ms; and (**d**) 21.4 ms.

The qualitative comparison of the modelling results and the images recorded for real splashes is presented in Figure 10. The example was chosen to show the best possible (white line in Figure 10) and worst possible matching of simulation and reality (red line in Figure 10).

A quantitative comparison of the simulation results and the analysis of the phenomenon based on the images recorded for all variants of the simulations are presented in Table 3. The relative error presented in this table was calculated from the formula:

(10) $$\mathrm{Relative}\;\mathrm{error}=\frac{\mathrm{value}\;\mathrm{from}\;\mathrm{the}\;\mathrm{experiment}\;-\;\mathrm{value}\;\mathrm{from}\;\mathrm{the}\;\mathrm{simulation}}{\mathrm{value}\;\mathrm{from}\;\mathrm{the}\;\mathrm{experiment}}\;$$

The analysis of the data from Table 3 shows that for both crown parameters (height and width), for both variants taking into account the variable drop diameter and variants taking into account the variable drop velocity, the smallest differences between the measured and simulated values were recorded for the variant d,v(0), i.e., for values read/calculated directly from the image analysis.

Generalising the analysis of differences between simulations and experiments in relation to the cavity, it should be noted that a greater impact was recorded for drop size variants. In this group of results, the smallest relative error was again obtained for the variant d,v(0). The change in the drop velocity across the entire assumed range did not significantly affect the differences between the cavity depth generated by the model and that recorded experimentally.

### 3.4. Stratification of Liquids

**Figure 11:**
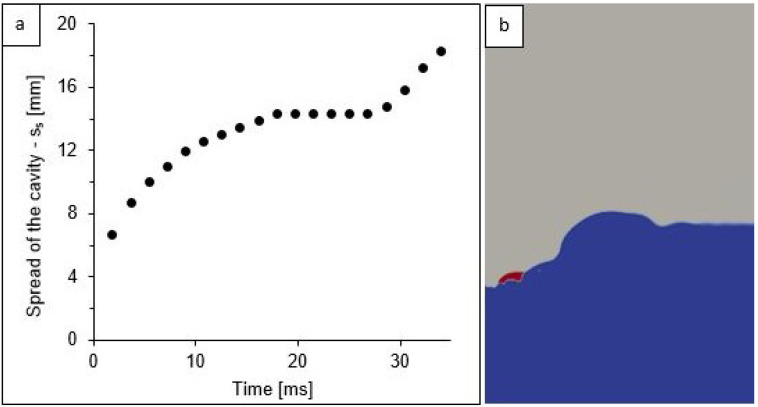
Cavity: (**a**) changes in the maximum cavity spread in a function of time for simulated variant d,v(0); (**b**) an example of cavity simulation in 30 ms for model d,v(0)—the colours signify: water (blue), petrol (red), and air (grey).

The compatibility of the d,v(0) model to the real measurements made it possible to use this simulation to describe aspects of the splash phenomenon that were not possible to observe during the experiment. From the d,v(0) model, the maximum cavity spread was read (this dimension was covered by the meniscus in the images of the real splash), and the analysis of the propagation of petroleum substances during the splash in a two-phase system was performed. The variability of the maximum cavity spread, measured at the liquid level, is shown in Figure 11.

The simulation d,v(0) revealed that, during cavity formation, three phases can be distinguished depending on the duration of the splash phenomenon (Figure 11a). In the first phase, from the moment of the impact of a drop on the surface up to about 16 ms, the cavity expanded rapidly. In the second phase, at about 17 ms, the cavity reached its greatest width, which remained constant until about 26 ms. A further increase in the width of the cavity occurred at the same time as the petrol “drop” re-collected (red dot in Figure 11b) to the centre of the system, and again with the formation of the jet. Therefore, this stage was considered as the end of cavity formation which, from that moment, turned into a circular wave propagating from the impact centre.

**Figure 12:**
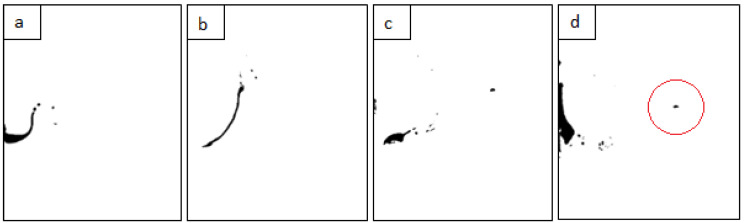
Simulation of the behaviour of a petrol drop causing a splash phenomenon in an immiscible fluid system: (**a**) spilling petrol drop on the inside of the crown and cavity, t = 1.8 ms; (**b**) petrol droplets detaching from the rim of the crown, t = 16.1 ms; (**c**) re-collection of most of the petrol to the impact centre, t = 26.7 ms; (**d**) the formation of the petrol into a secondary splash form, the so-called jet, t = 33.9 ms. The red circle marks the petrol droplet that detached from the crown spike and landed farthest from the centre of the impact.

The initial stages of the phenomenon, during which petrol formed a layer between air and water and when petrol droplets detached from the crown, are shown in Figure 12a,b. The later stages, during which petrol returned to the centre of the system and next the jet was created, are presented in Figure 12c,d. Another way of illustrating the behaviour of petrol is shown in Figure 3a, Figure 7b, Figure 9b and Figure 11b.

**Figure 13:**
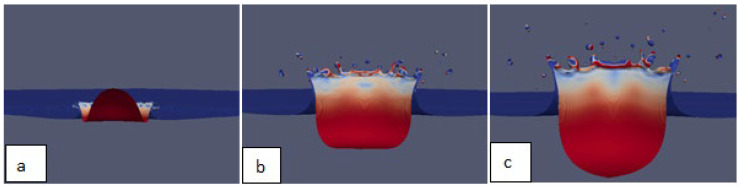
Visualisation of selected times of the 3D simulation of the multiphase splash phenomenon, depicted from the drop impact centre: (**a**) 0.7 ms; (**b**) 1.8 ms; (**c**) 3.2 ms time.

A model with fine-tuned parameters was finally used for a true 3D simulation of the multiphase splash phenomenon. The figures below show crown formation under the impact of the petrol drop (Figure 13a) and the growth of the crown with the simultaneous spread of droplets (Figure 13b,c). The splash phenomenon was shown from the perspective of the centre of the liquid system. ParaView images show the phase boundary, with the colours representing different fractions: the blue colour represents the water and the red colour represents the falling petrol drop that spreads on the inner walls of the cavity in the later phases.

## 4. Discussion

### 4.1. Analysis of the Resulting Forms

When analysing the graphs presented in Figure 4, it is worth noting that the crown height and cavity depth tended towards zero (at 34 ms), while the values of crown width in the observed time interval kept increasing. In order to explain this well, it is necessary to analyse the course of the phenomenon shown in Figure 5.

As a result of the impact of the drop on the water surface, a crown and a cavity were formed. Both these forms reached their maximum, however, at different times: the height of the crown after about 8 ms and the cavity depth after about 17 ms. Next, the surface of the liquid (in our case, water) tended to level out (crown height and cavity depth approached zero). This moment may be considered the end of the existence of the primary forms. However, before this happened, a secondary form, i.e., the vertical jet, had already begun to form.

The dimensions and upward velocity of the jet depend on the experimental conditions. As evidenced by Morton et al. [14], for low values of We and Fr (both less than 100), bubble entrapment occurs during cavity collapse. The characteristic cavity shape changes from conical to cylindrical as a result of the capillary wave that forms during the initial stages of cavity growth and then propagates down the walls of the expanding cavity. The trapping of the bubble under the bottom of the cavity simultaneously results in the formation of a thin, high speed upward jet. However, there is an upper limit to the bubble entrainment since the maximum cavity size increases with Fr and We, while the capillary wave velocity decreases. Therefore, if the wave front fails to reach the cavity base before collapse occurs, a thick Rayleigh jet is formed. As shown by Morton et al. [14], for a drop impact at higher values of We and Fr (above 200), the cavity collapses without forming a bubble, instead, the thick jet is formed, as in our experiments.

In Figure 4, the recorded height of the jet is marked at the last analysed moment. The fact that the jet was noticed for the first time at 34 ms does not mean that it was not formed earlier. However, identifying it at an earlier stage was not possible because it formed at the bottom of the cavity before the liquid surface levelled out. This was confirmed by the values of the height of the jets shown in Figure 7 and Figure 9, wherein some variants of the simulation, there were jets with a lower height than the still observable cavity depth. This is particularly evident in the image of the example simulation shown in Figure 9b.

The crown width was recorded only up to about 12 ms, which is due to the fact that this was the last moment of the existence of the “classical” crown. Up to that point, the crown was well formed and its width could be clearly determined. After about 12 ms, the crown began to fall and lose its shape, forming a drifting wave of water (Figure 5).

The course of cavity formation was slightly different. The changes in cavity depth (Figure 4) can be separated into three phases: increasing depth, maintaining constant maximum depth, and finally decreasing depth. Each of these phases lasted for about one-third of the duration of the phenomenon, from the moment of the drop impact to the disappearance of the cavity.

The above-mentioned meniscus occurring at the vessel wall made it impossible to measure the cavity spread, which was equal to the width of the crown base. Determination of this dimension was only possible based on the simulation results (Figure 11a).

Crown splash has previously been a fairly common object of study [7,51]. Both from the experiments and from the numerical simulations, the authors parameterised its size, determined the spike frequency, and/or the number of detaching satellite droplets. Beczek et al. [52] observed differences in crown formation during water drop impact onto a thin water film formed on a smooth glass surface and on the surface of saturated soil. Liang et al. [21] discussed the influence of Weber and Reynolds numbers on the crown dimensions and showed that the crown height increases with the increasing of the Weber number, but the Reynolds number has only a small effect on it, while the crown diameter is independent of these two non-dimensional numbers. The crown formation using drops of different fluids on various film depths of the same fluids was reported by Van der Wal and Berger [53]. Rioboo et al. [54] investigated the crown behaviour at sufficiently high-impact velocities on a thin film of liquid using silicon oils and a water–glycerol mixture. Krechetnikov and Homsy [55] discussed the crown-forming instability problem during water or milk drop splash on a thin film of the same liquid. The above-mentioned results are difficult to compare due to the fact that they were determined by specific experimental conditions, related in particular to the type of liquid used, the nature of the impact, or the energy of the falling drop. Nevertheless, the previously published data confirm the regular and repeatable course of the splash phenomenon.

A parametrisation of the cavity shape by determining the maximum cavity dimensions and cavity collapse was presented in [8]. The authors proved that the depth of the cavity depends on the Weber number, i.e., directly on the drop velocity, assuming a constant size (100-micrometres in their case). Furthermore, based on the theoretical analysis and the experimental observations, Fedorchenko and Wang [7] showed that the maximum cavity radius and cavity collapse time depend on both the Froude number and the dimensionless capillary length ($l_{c}=\frac{\sqrt{\frac{2\sigma }{\rho g}}}{d}$), depending on the size of the falling drop. As proved by Bouwhuis et al. [56] in an experiment of the microdrops train impact into a deep pool and based on the 2D Rayleigh equation, the cavity shape is a parabola, which translates downward with a constant velocity, and this velocity depends on the ratio of the drop diameter to the drop distance in the train. In addition, as concluded by the authors, the expansion of the cavity can be described as a purely inertial mechanism, except for the collapse region, which is due to capillary forces.

### 4.2. Analysis of the Simulation of Changes in the Size of the Drop and Its Impact Velocity

As already mentioned, the estimation of the kinetic energy of the falling drop could be subject to error due to the uncertainty in the reading of the drop diameter and its velocity. The uncertainty for both of these quantities was due to the fact that the phase boundary (petrol/air) in the recorded images was blurred. Since the scale of the blurring was within ±3 pixels, simulations were run for all such cases (Table 2).

Analysing the simulation results, it should be noted that the error in reading the drop size affected the results to a much greater extent (Figure 6) than the error in estimating the impact velocity (Figure 8). Considering the kinetic energy formula ($E_{k}=\frac{mv^{2}}{2}$), the velocity is squared, so intuitively, any error in the drop velocity measurement should affect the result more than an error in the drop size measurement. However, it is important to realise that the velocity is calculated from the distance that the falling drop travels in a given time—in practice, it is the difference between the location of a particular point (of a drop) at the beginning of the measurement and at its end. Thus, the addition (or subtraction) of one, two, or three pixels to this distance yielded a small error, which does not cause a large variation in the calculated velocity (maximum variation 2.4%). In contrast, changing the drop size by three pixels for the extreme variants affected this parameter by up to 9.1%.

In terms of the effect of changes in drop kinetic energy on the jet formation (Figure 7 and Figure 9), it can be observed that the bigger the difference between the values constituting the input data to the simulation, the bigger the difference in the formation time and jet size. In this case, a smaller variation in results was also observed for variants with a variable drop velocity than for variants with a variable drop diameter. However, it should be noted that this part of the simulation (devoted to secondary forms) was run for a very short time; hence, it is not possible to explicitly state what the dynamics of the changes would be in subsequent time steps.

The energy of a falling drop is considered to be one of the factors with a dominant influence on the course of splashing phenomena in liquid systems [6,17,23,57]. The published data prove that a change in this parameter can significantly affect the dynamics of the phenomenon, as well as the size, shape, and type of splash forms [11,13,37,58]. As concluded by Sochan et al. [13] in their analyses of 11 energy variants in three different single-phase systems, different, even very different, splash forms were formed as the drop energy increased. In extreme cases, the splash form was a small circular wave (low-energy variant) or a closed crown, so-called “dome” (high-energy variant). In our simulations, the splashes induced by drops with seven different velocities differed in a much less pronounced manner, which was an effect of the already emphasised narrow ranges of the velocities considered. Nevertheless, the differences in the form dimensions exceeded even 50%, although the resulting splash forms were always the same and the duration of the phenomenon was similar (about 35 ms until the momentary equilibration of the liquid surface after the original splash forms dissipated).

### 4.3. Validation of the Model

In order to indicate which results from the 13 simulations of the phenomenon were the most accurate, they were compared with the results of measurements recorded with the high-speed camera. The results of these comparisons are presented in Table 3. Summarising the presented data, it can be concluded that in all analysed cases, the best fit (when the median difference between the simulated and the experimental values is as close as possible to zero) was obtained for the d,v(0) variant, i.e., for the actual reading of the drop value. The graphical representation of the interfacial profile from the simulation d,v(0) in the real images of the splash (white line in Figure 10) showed the greatest agreement of the obtained splash forms. Therefore, this variant was considered in further analyses.

The comparisons prove the significance of the influence of overestimation/underestimation of the considered parameters even by one pixel on the result of numerical calculations. Hence, the correct determination of the input data to the Navier–Stokes equations is a key difficulty in numerical modelling.

### 4.4. Description of the Behaviour of Both Liquids (Petrol and Water) during a Splash

The validation of the numerical model based on the experimental data proved that the application of the VOF method to describe the splash of immiscible liquids gave very good results. The construction of numerical models provides an opportunity not only to describe new experiments physically, but also parameterise previous studies more accurately. Such a comparison was made by e.g., Reijers et al. [37], who created a splash model for a broad range of Weber numbers and impact angles, based on the experiment reported by Gielen et al. [8], where good qualitative and quantitative agreement of the results was achieved. Similarly, Guilizzoni et al. [20] performed numerical simulations of double synchronised water drop impacts using the InterFoam solver of the OpenFOAM package, which they verified with the experimental data presented in [19]. Comparison of the results proved good agreement, particularly in the inertial phase (leading to the maximum crater depth). In contrast, Bouwhuis et al. [56] provided an important caveat that comparing experimental and numerical simulation results would only work if the influence of air was not very significant. They noted that the experimental cavity width caused by the impact of a train of water microdroplets was smaller than the width in the simulations; this was due to the influence of the streaming air, which was neglected. Nevertheless, the authors concluded that their simulation method was an efficient way to simulate the drop train impact with respect to the depth of the cavity, neglecting the influence of air.

Based on experimental conditions specified by Chen et al. [59], Ray et al. [4] developed a numerical simulation of drop impact with the liquid interface. It is worth mentioning that they analysed a system of immiscible liquids in which the impact of a small drop with low velocity induced a coalescence phenomenon. The authors proposed a mechanism by which a transition from complete coalescence to partial coalescence occurs. Their model implemented a 2D description, assuming axial symmetry of the phenomenon. In contrast, in our analysis, the higher dynamics of the system resulted in a splash and thus a crown with detaching droplets; hence, our comparison involves 3D modelling.

The proper simulation of the phenomenon provided a three-dimensional view of the splash and facilitated analysis of aspects that were difficult or even impossible to measure experimentally. Even in the case of the crown and cavity dimensions, which are relatively simple to calculate and are often determined by researchers, an accurate simulation improves the reliability of the result, since it does not leave doubts arising from a blurred contour of the form or a partially obscured image by the meniscus. Moreover, the model gives the possibility of analysing the behaviour of both liquids during the splash, i.e., monitoring which liquid was ejected and to what extent. Such information is important when petrol contamination is spreading, and helps to understand and predict the behaviour of natural environmental systems including water and air.

As evidenced by the images shown (Figure 12a–c), most of the petrol from the drop causing the splash spread on the inner walls of the cavity. This is related to the fact that petrol and water are immiscible and, in addition, petrol has a lower density than water. Other cases of drop behaviour in a liquid system are presented in [17]. The authors obtained different forms of splash and mechanisms of mixing of the liquids, which they named, for example, lens, jellyfish, halo, or chaos. The trapping of an air bubble below the surface is another frequently observed phenomenon accompanying the impact of a drop on a deep liquid layer [6,14,15]. As inferred in [15], the formation of this form is dependent not only on the velocity of the drop, but also on its shape at the time of contact with the liquid surface.

In our simulations, as the cavity depth began to reduce, water and petrol that had drained from the walls began to erupt in a jet form. Figure 12d shows the moment when the formation of the jet began, with the top of the jet formed by the collected petrol. After the splash phenomenon ended, when the liquids in the vessel had levelled out, the greater part of the petrol was collected centrally at the point of the drop impact, while its smaller part was ejected as satellite droplets that detached from the crown and carried the contamination up to a distance estimated as several centimetres. The process of droplet detachment from the crown spikes is shown in Figure 13b,c, while the location of the “landing” of a petrol droplet is indicated in Figure 12d.

As reported by Liow [9] for low impact velocity (We below 10), a gently deposited drop may float momentarily before it collapses into the bath due to gravity. At higher values of Fr and We, the falling drop coalesces on the impact with the liquid surface. The coalescence may take different forms: (i) total coalescence occurs when the entire drop merges with the underlying reservoir, (ii) partial coalescence arises when only a fraction of the drop coalesces, leaving behind a smaller drop that is ejected from the bath and may bounce several times before undergoing itself to total coalescence [60]. As also described by Thoroddsen and Takehara [60], the coalescence process may then take place in a cascade, where each step generates a smaller drop, whereby the cascade does not proceed ad infinitum due to viscous effects, which begin to be important for the smaller drops.

Yakhshi-Tafti et al. [22] found that for low values of We (about 20), a droplet striking the surface of a system of immiscible liquids can withstand the impact and maintain its spherical shape at the air-liquid interface while the liquid surface deforms similar to an elastic membrane and causes the droplet to oscillate on its surface. The duration of this oscillation does not exceed 70 ms, due to the strong viscous damping. Rein [5] studied the boundaries between coalescence and splashing phenomena and showed that as We and Fe increase simultaneously, the regime of coalescence shifts towards the splashing regime. Lhuissier et al. [10] showed that as the Weber number increases further, liquid sheet and drop fragmentation can occur and the number of daughter droplets increases. They also found that for an impact at We = 1250, not all daughter droplets remain in the liquid, but some are ejected above the surface into the air. Similarly, Murphy et al. [11] deduced that, depending on the thickness of the oil film, an estimated number of tens of droplets and thousands of smaller droplets (fine droplet aerosol) may be ejected. As stated by the authors, the impact of raindrops on the surface of a contaminated liquid (e.g., after a fuel spill) is the mechanism for transferring contaminants from the sea surface to the atmosphere and forming an oily marine aerosol.

The above conclusions are consistent with our results, where for the impact at We = 986, the crown formation phenomenon was dynamic and involved the detachment of many secondary droplets from its spikes. It should also be emphasised that the simulation of the phenomenon showed that the detaching droplets consisted not only of water but were mostly a “mixture” of water and petrol, or petrol alone in the case of the finest droplets (Figure 13b,c).

## 5. Conclusions

Using experimental results, this study successfully validated the multiphaseInterFoam solver, a part of the OpenFOAM CFD software suite. The validated CFD multiphase solver ensured the correct 3D simulation of the splash phenomenon during the existence of primary forms (crown and cavity).

Correctly defining and determining the initial values is a key modelling difficulty. Even a slight underestimation/overestimation (of the order of 1%) of the incident drop velocity and size has a significant impact on the calculated intensity and dynamics of the splash phenomenon. The simulation variants performed unambiguously indicate that for small changes in the diameter and impact velocity of the incident petrol drop, it is the diameter that has a greater impact on the resulting splash.

The numerical simulations present a three-dimensional view of the impact phenomenon and provide a valuable addition to the experimental results. Based on the simulations performed, the width of the cavity (equal to the width of the crown base), the moment of cavity disappearance, the moment of jet formation (still below the water level), and the height of the jet were determined, which were not available in the experiments due to the existing meniscus partially obscuring these splash forms. Moreover, the simulation of the splashing of immiscible liquids provided information on the spreading of petroleum substances. It showed that most of the petrol from the drop causing the splash was located centrally as a spot, and a smaller part was ejected as small droplets over a distance estimated as several centimetres. Until now, such an inference has not been possible without specific chemical analyses.

## Figures and Tables

**Table 1:** Basic characteristics of the investigated fluids [13].

Fluid Property (in 20 °C)	Water	Petrol	Air
Density (kg∙m^−3^)	998.2	748.0	1.2
Kinematic viscosity (m^2^∙s^−1^)	1.0 × 10^−6^	0.4 × 10^−6^	14.9 × 10^−6^
Surface tension for a fluid-air interface (mN∙m^−1^)	72.94	28.42	-
Interfacial tension (mN∙m^−1^)	35.0		

**Table 2:** Symbols and input data for 13 variants of simulation.

Modelling Variant		Drop Properties	
		Diameter (d) (mm)	Velocity (v) (m∙s^−1^)
d(−3)	−3 pixels	3.00	3.37
d(−2)	−2 pixels	3.10	
d(−1)	−1 pixel	3.20	
d,v(0)	Read from the recorded frame	3.30	
d(+1)	+1 pixel	3.40	
d(+2)	+2 pixels	3.50	
d(+3)	+3 pixels	3.60	
v(−3)	−3 pixels	3.30	3.29
v(−2)	−2 pixels		3.32
v(−1)	−1 pixel		3.34
d,v(0)	Read from the recorded frame		3.37
v(+1)	+1 pixel		3.39
v(+2)	+2 pixels		3.42
v(+3)	+3 pixels		3.44

**Table 3:** Relative error calculated as the ratio of the median of the difference between the measured and simulated value to the measured value. Variants for which the absolute value was the smallest are underlined.

	**Drop Size Variants**						
	**d(−3)**	**d(−2)**	**d(−1)**	**d,v(0)**	**d(+1)**	**d(+2)**	**d(+3)**
crown height	0.20	0.15	0.10	−0.03	−0.14	−0.22	−0.43
crown width	0.10	0.09	0.04	0.00	−0.01	−0.01	0.01
cavity depth	0.10	0.06	0.03	0.01	−0.02	−0.03	−0.06
	**Drop Velocity Variants**						
	**v(−3)**	**v(−2)**	**v(−1)**	**d,v(0)**	**v(+1)**	**v(+2)**	**v(+3)**
crown height	0.05	−0.03	−0.03	−0.03	−0.06	−0.07	−0.08
crown width	0.04	0.02	0.02	0.00	0.01	0.01	0.01
cavity depth	0.02	0.03	0.02	0.01	0.01	0.01	0.01
